# Lipoprotein(a) Reflects Baseline Lipid Phenotype but Does Not Predict Long-Term Cardiometabolic Risk in Apparently Healthy Women

**DOI:** 10.3390/metabo16060390

**Published:** 2026-06-04

**Authors:** Seokhwan Yoon, Minjung Kang, Hyun Suk Yang

**Affiliations:** 1 Department of Cardiovascular Medicine, Konkuk University Medical Center, Seoul 05030, Republic of Korea; yseokh123@gmail.com; 2 International Healthcare Center, Konkuk University Medical Center, Seoul 05030, Republic of Korea; 20120635@kuh.ac.kr

**Keywords:** lipoprotein(a), cardiometabolic disease, dyslipidemia, women, longitudinal cohort, lipid phenotype, risk prediction

## Abstract

**Highlights:**

**What are the main findings?**

• In apparently healthy women with a median age of 41 years, elevated Lp(a) (≥50 mg/dL) was associated with higher total cholesterol and LDL-C at baseline but was not associated with long-term incident cardiometabolic disease over a median follow-up of 12.6 years.
• The null association was consistent across all analytical approaches, including multivariable Cox models, a predefined ≥10-year follow-up subgroup, and sensitivity analyses treating Lp(a) as a continuous variable.

**What are the implications of the main findings?**

• Lp(a) reflects an adverse baseline lipid phenotype in apparently healthy younger women but does not independently predict future cardiometabolic risk, suggesting limited utility for cardiometabolic risk prediction in this population.
• Clinicians should interpret Lp(a) levels in a population- and outcome-specific context; its utility for cardiometabolic risk prediction may be most relevant for ASCVD risk assessment in older or postmenopausal women rather than for upstream outcomes in younger populations.

**Abstract:**

Background/Objectives: Lipoprotein(a) [Lp(a)] is an established risk-enhancing biomarker for atherosclerotic cardiovascular disease (ASCVD) and is increasingly incorporated into preventive risk assessment. However, whether Lp(a) predicts long-term cardiometabolic disease (CMD) beyond its associations with lipid parameters in apparently healthy women remains unclear. Methods: We retrospectively analyzed 559 women (median age 41 [36–46] years) who underwent comprehensive health check-ups with baseline Lp(a) measurements. After excluding those with baseline CMD, ASCVD, or insufficient follow-up, 387 women formed the primary longitudinal cohort. Participants were stratified by Lp(a) level (<50 vs. ≥50 mg/dL). Incident composite CMD, defined as new-onset hypertension, diabetes mellitus, or dyslipidemia, was assessed using Kaplan–Meier analysis, Cox proportional hazards models, and sensitivity analyses treating Lp(a) as a continuous variable and restricting the analysis to participants with ≥10 years of follow-up. Results: At baseline, elevated Lp(a) (≥50 mg/dL) was associated with higher total cholesterol and LDL-C and a greater prevalence of dyslipidemia, with a modest Lp(a)–LDL-C correlation (ρ = 0.24, *p* < 0.001). Over a median follow-up of 12.6 years, CMD incidence did not differ between Lp(a) groups (33.3% vs. 35.3%, *p* = 0.907). Lp(a) was not associated with incident CMD in multivariable Cox models (adjusted HR 0.81, 95% CI 0.49–1.34), with consistent findings in the ≥10-year follow-up subgroup (*n* = 224) and in continuous-variable sensitivity analyses. Conclusions: In apparently healthy women, elevated Lp(a) reflects an adverse baseline lipid phenotype but does not independently predict long-term incident CMD. These findings suggest that the clinical utility of Lp(a) may be context-dependent, with its predictive value primarily limited to ASCVD risk assessment rather than broader cardiometabolic risk prediction in this population.

## 1. Introduction

Lipoprotein(a) [Lp(a)] has emerged as an important cardiovascular risk factor and is increasingly recognized as a risk-enhancing biomarker in contemporary prevention strategies. Elevated Lp(a) levels are largely genetically determined and have been causally linked to atherosclerotic cardiovascular disease (ASCVD) in multiple large-scale Mendelian randomization studies and prospective cohorts [1,2,3]. Both the 2025 ESC/EAS focused update and the 2026 ACC/AHA dyslipidemia guideline recommend Lp(a) measurement at least once in all adults for ASCVD risk assessment [4,5].

Despite these advances, important sex-specific considerations remain underexplored. In women, Lp(a) levels are influenced by endogenous estrogen, which suppresses hepatic Lp(a) production through inhibition of *LPA* gene transcription, resulting in lower concentrations during the reproductive years [6,7]. Following menopause, Lp(a) levels rise substantially [8], potentially amplifying cardiovascular risk during a period when conventional risk models already tend to underestimate future events. Nevertheless, evidence supporting the role of Lp(a) as a predictor of long-term cardiometabolic outcomes in apparently healthy women across the reproductive lifespan remains limited.

Furthermore, most existing studies on Lp(a) have focused on established ASCVD outcomes or mortality. In relatively young and apparently healthy populations, the incidence of such hard endpoints is inherently low, limiting their utility for risk stratification at early disease stages. In this context, broader cardiometabolic conditions—including hypertension, diabetes mellitus, and dyslipidemia—represent earlier and more frequent manifestations of metabolic dysregulation and may serve as more sensitive outcome measures in preventive settings. Identifying whether Lp(a) contributes to these upstream risk trajectories is therefore of particular clinical relevance.

In this study, we aimed to investigate whether baseline Lp(a) levels are associated with the long-term incidence of cardiometabolic disease (CMD) in a well-defined cohort of apparently healthy women undergoing a comprehensive health check-up program. We additionally sought to characterize the relationship between Lp(a) and baseline lipid parameters in this population.

## 2. Materials and Methods

### 2.1. Study Population

**Figure 1:**
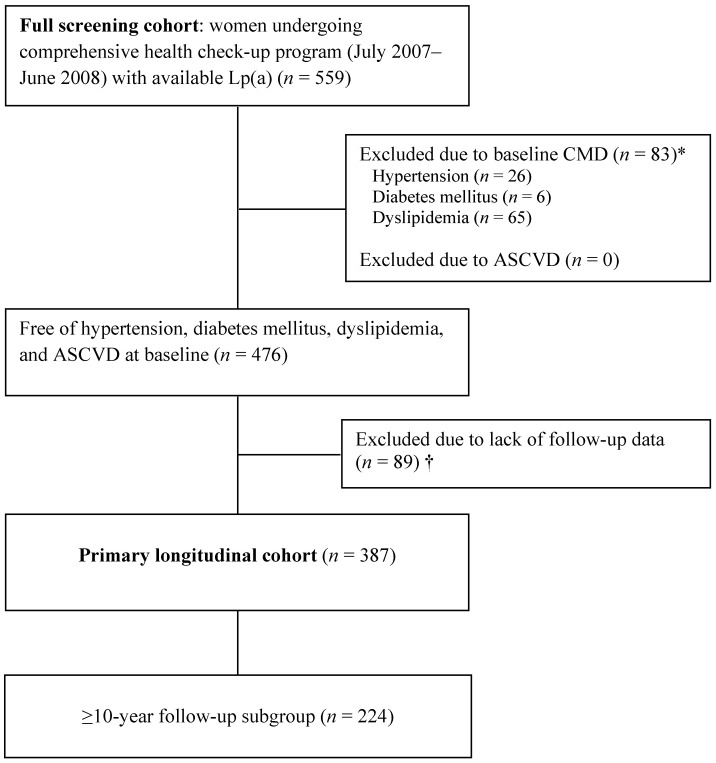
Study flow diagram of the study population. Flow diagram showing the selection of the study population from the full screening cohort (*n* = 559) to the primary longitudinal cohort (*n* = 387) and the ≥10-year follow-up subgroup (*n* = 224). * Baseline CMD was defined as hypertension, diabetes mellitus, or dyslipidemia. Categories were not mutually exclusive. † After the index health check-up, no clinical or laboratory data were available to ascertain incident CMD, ASCVD, or death. Abbreviations: Lp(a), lipoprotein(a); CMD, cardiometabolic disease; ASCVD, atherosclerotic cardiovascular disease.

This single-center retrospective cohort study included women who underwent a comprehensive health check-up program at Konkuk University Medical Center between July 2007 and June 2008 and had available baseline Lp(a) measurements. The program included a standardized health questionnaire, anthropometric measurements, blood pressure assessment, fasting laboratory testing, imaging studies, and physician review of the examination results. The initial screening cohort comprised 559 women. Individuals with baseline CMD, defined as hypertension, diabetes mellitus, or dyslipidemia, or with established ASCVD at the index visit, were excluded. Participants for whom no clinical or laboratory data were available after the index health check-up to ascertain incident CMD, ASCVD, or death were excluded. The final primary longitudinal cohort consisted of 387 women who were free of CMD and ASCVD at baseline and had available follow-up data. Among them, 224 women with a follow-up duration of at least 10 years were included in a predefined ≥10-year follow-up subgroup analysis (Figure 1).

### 2.2. Clinical and Laboratory Measurements

Baseline clinical and laboratory data were obtained at the index health check-up visit. Demographic and clinical variables included age, body mass index (BMI), and smoking status. Blood samples were collected after an overnight fast and analyzed using standardized laboratory methods. Measured parameters included Lp(a), total cholesterol, low-density lipoprotein cholesterol (LDL-C), high-density lipoprotein cholesterol (HDL-C), triglycerides, and fasting glucose.

Lp(a) was measured by a central reference laboratory (Seoul Clinical Laboratories, Seoul, Republic of Korea) using a turbidimetric immunoassay (TIA) and reported in mg/dL, consistent with routine clinical practice during the study period. Values below the assay lower limit of detection (LoD) of 8 mg/dL were assigned a value of 4 mg/dL (LoD/2) for analytical purposes, consistent with standard practice for left-censored laboratory data; this applied to 60 women (15.5%) in the primary longitudinal cohort. As all such values fell well below the predefined categorical threshold of 50 mg/dL, this substitution had no impact on the primary group classification or Cox regression analyses.

### 2.3. Definitions of Baseline CMD and Outcomes

Baseline CMD was defined as the presence of hypertension, diabetes mellitus, or dyslipidemia at the index health check-up visit. Hypertension was defined as a prior clinical diagnosis, use of antihypertensive medications, or physician-documented hypertension in the electronic medical records. Isolated elevated blood pressure measurements obtained at a single health check-up visit were not considered diagnostic. Diabetes mellitus was defined as a prior diagnosis, use of glucose-lowering medications or insulin, physician-documented diabetes, or glycated hemoglobin (HbA1c) ≥ 6.5% at the time of assessment. Dyslipidemia was defined as a prior diagnosis, use of lipid-lowering medications, physician-documented dyslipidemia, or abnormal lipid levels, including LDL-C ≥ 160 mg/dL, triglycerides ≥ 200 mg/dL, or HDL-C < 40 mg/dL.

The same criteria were applied to define incident CMD during follow-up. The primary outcome was incident composite CMD, defined as the new onset of at least one of these conditions. ASCVD and all-cause mortality were assessed as secondary outcomes.

### 2.4. Follow-Up

Participants were followed from the date of the index health check-up through subsequent clinical encounters recorded in the electronic medical records, including outpatient visits, laboratory measurements, and physician-documented diagnoses.

Follow-up duration was calculated from the index date to the first occurrence of CMD or the last available clinical visit. For the composite CMD outcome, time-to-event was defined as the time to the first occurrence of any component (hypertension, diabetes mellitus, or dyslipidemia). Participants without incident CMD were censored at the time of their last follow-up. A subgroup analysis was performed in participants with a follow-up duration of at least 10 years.

### 2.5. Statistical Analysis

Continuous variables are presented as median [interquartile range], and categorical variables as counts and percentages. Comparisons between groups according to baseline Lp(a) levels were performed using the Mann–Whitney U test for continuous variables and the chi-square test or Fisher’s exact test for categorical variables, as appropriate. Spearman’s rank correlation coefficients were used to assess associations between Lp(a) and metabolic parameters.

The primary categorical analysis used a predefined Lp(a) threshold of 50 mg/dL, consistent with the cutoff recognized as a significant risk enhancer or high-risk threshold across contemporary cardiovascular prevention guidelines [4,5,6,9]. The cumulative incidence of CMD was evaluated using Kaplan–Meier survival analysis, and differences between groups were assessed using the log-rank test. Associations between Lp(a) and incident CMD were examined using Cox proportional hazards regression models in four sequential steps: Model 1, unadjusted; Model 2, adjusted for age; Model 3, adjusted for age and BMI; and Model 4, additionally adjusted for LDL-C. The proportional hazards assumption was verified using Schoenfeld residuals. In addition to categorical analysis, Lp(a) was analyzed as a continuous variable in sensitivity analyses. A two-sided *p*-value < 0.05 was considered statistically significant. All statistical analyses were performed using R software (version 4.3.2).

## 3. Results

### 3.1. Baseline Characteristics According to Lp(a) Levels

**Figure 2:**
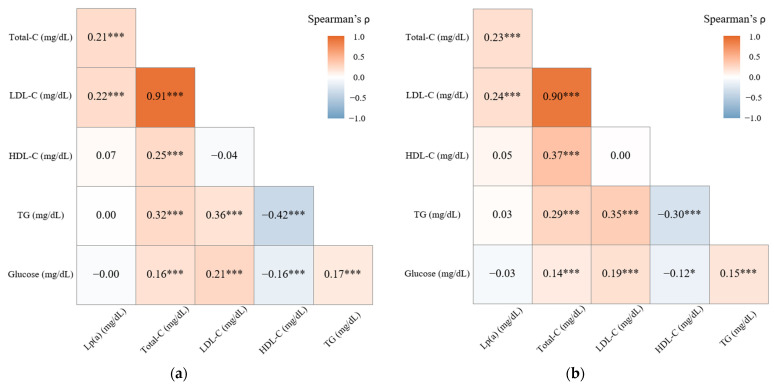
Correlation of baseline lipoprotein(a) with lipid and metabolic parameters. (**a**) Full screening cohort (*n* = 559). (**b**) Primary longitudinal cohort (*n* = 387). Heatmaps showing Spearman’s rank correlation coefficients (ρ) between baseline lipoprotein(a) [Lp(a)] and lipid and metabolic parameters. Color intensity represents the strength and direction of correlation. * *p* < 0.05, *** *p* < 0.001. Abbreviations: Lp(a), lipoprotein(a); Total-C, total cholesterol; LDL-C, low-density lipoprotein cholesterol; HDL-C, high-density lipoprotein cholesterol; TG, triglycerides.

Among the full screening cohort (*n* = 559), 86% of participants had Lp(a) < 50 mg/dL, while 14% had elevated Lp(a) ≥ 50 mg/dL. The median age was 41 [36–46] years, with no significant difference between groups. Women with elevated Lp(a) exhibited a more adverse lipid profile at baseline, including higher total cholesterol and LDL-C levels (both *p* = 0.001) and a greater prevalence of dyslipidemia (20.8% vs. 10.2%, *p* = 0.012). No significant differences were observed in BMI, triglycerides, HDL-C, fasting glucose, or smoking status between groups (Table 1). Lp(a) showed modest positive correlations with total cholesterol (ρ = 0.23, *p* < 0.001) and LDL-C (ρ = 0.24, *p* < 0.001), with no significant correlation with HDL-C, triglycerides, or fasting glucose (Figure 2a).

In the primary longitudinal cohort (*n* = 387), restricted to participants free of baseline CMD and ASCVD, similar patterns were observed. Women with elevated Lp(a) had higher total cholesterol and LDL-C levels (Table 2), with modest positive correlations with total cholesterol (ρ = 0.21, *p* < 0.001) and LDL-C (ρ = 0.22, *p* < 0.001) (Figure 2b).

### 3.2. Association Between Lp(a) and Incident CMD

In the primary longitudinal cohort (*n* = 387), the median follow-up duration was 12.6 years, and 57.9% of participants were followed for more than 10 years. No ASCVD events or deaths occurred during follow-up.

The cumulative incidence of composite CMD was 33.6% (*n* = 130), with dyslipidemia as the predominant component (31.5%), followed by hypertension (7.8%) and diabetes mellitus (4.1%). The incidence of composite CMD did not differ between Lp(a) groups (33.3% vs. 35.3%, *p* = 0.907) (Table 2). Similar findings were observed in the ≥10-year follow-up subgroup (49.2% vs. 48.4%, *p* = 1.00) (Supplementary Table S1).

**Figure 3:**
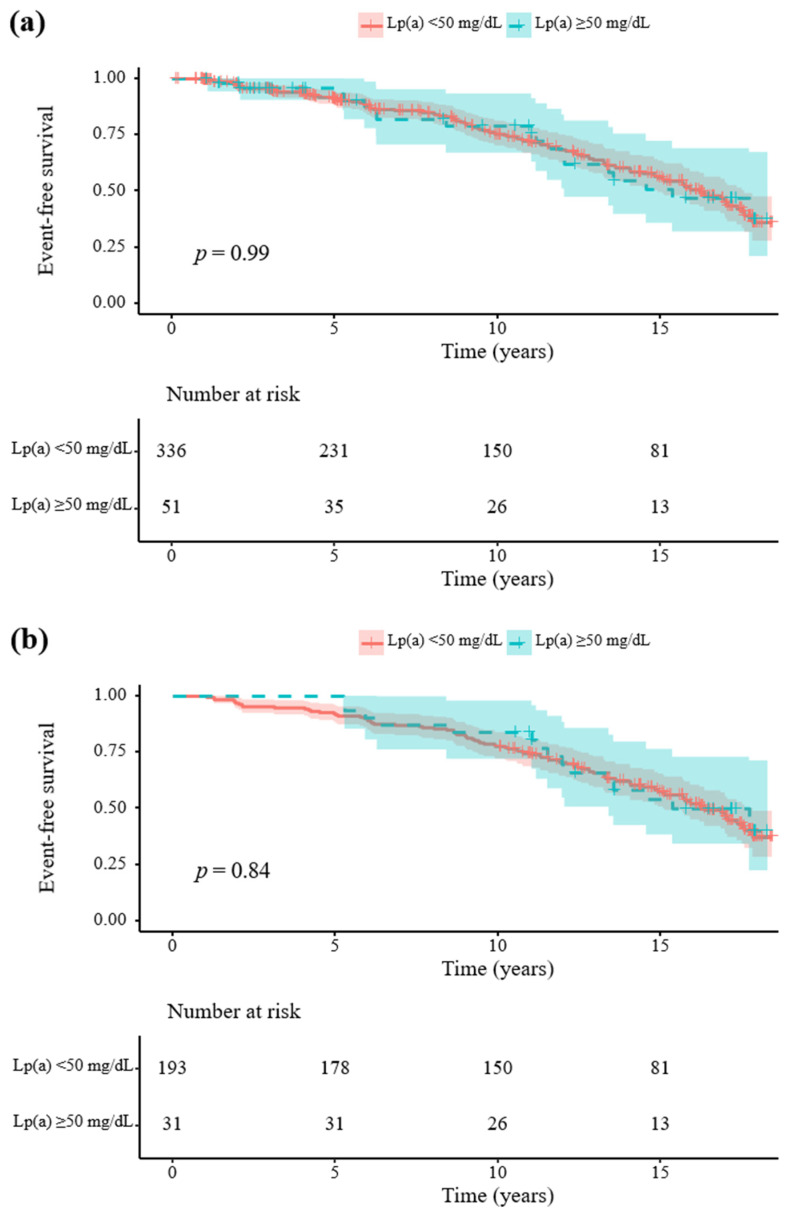
Kaplan–Meier curves for incident composite cardiometabolic disease according to baseline Lp(a) levels. (**a**) Primary longitudinal cohort (*n* = 387). (**b**) Subgroup with ≥10-year follow-up (*n* = 224). Composite cardiometabolic disease was defined as the occurrence of hypertension, diabetes mellitus, or dyslipidemia during follow-up. Shaded areas represent 95% confidence intervals, and tick marks indicate censored observations. Abbreviations: Lp(a), lipoprotein(a).

Kaplan–Meier analysis demonstrated overlapping cumulative incidence curves between groups, with no significant difference by log-rank test (Figure 3a). In Cox proportional hazards models, elevated Lp(a) (≥50 mg/dL) was not associated with incident CMD in any model, from the unadjusted model (hazard ratio [HR] 1.00, 95% confidence interval [CI] 0.61–1.64) through the fully adjusted model (HR 0.81, 95% CI 0.49–1.34) (Table 3). These findings were consistent in the ≥10-year follow-up subgroup (Figure 3b and Table 3). In sensitivity analyses treating Lp(a) as a continuous variable, no significant association with incident CMD was observed across any model in either cohort (Supplementary Table S2). To further examine the individual components of the composite endpoint, separate Kaplan–Meier analyses were performed for incident hypertension, diabetes mellitus, and dyslipidemia. No significant differences in event-free survival were observed according to baseline Lp(a) category for hypertension (log-rank *p* = 0.56), diabetes mellitus (log-rank *p* = 0.11), or dyslipidemia (log-rank *p* = 0.69) (Supplementary Figure S1).

## 4. Discussion

The principal finding of this study is that elevated Lp(a) in apparently healthy women of reproductive age was associated with an adverse baseline lipid phenotype—specifically higher total cholesterol and LDL-C and a greater prevalence of dyslipidemia—but did not independently predict the long-term incidence of CMD. This null association was robust across all analytical approaches, including multivariable Cox regression, continuous-variable sensitivity analyses, and a predefined subgroup with at least 10 years of follow-up.

This dissociation between baseline lipid co-clustering and long-term cardiometabolic risk is consistent with emerging evidence that Lp(a)’s predictive utility is highly context-dependent—shaped not only by outcome domain, but also by the baseline risk level and hormonal status of the population studied [10]. In one of the most comprehensive prospective studies of Lp(a) and type 2 diabetes risk conducted to date, Mora et al. followed 26,746 initially healthy US women for 13 years and found an inverse association between Lp(a) and incident diabetes, with women in the highest Lp(a) quintile experiencing approximately 22% lower diabetes risk—a direction entirely opposite to Lp(a)’s proatherogenic properties [11]. Similarly, Paige et al. reported in a prospective cohort study and meta-analysis that individuals with very low Lp(a) concentrations had a higher risk of incident type 2 diabetes [12]. Although we did not observe a statistically significant association between Lp(a) and incident diabetes, it is noteworthy that no cases of incident diabetes occurred among women with elevated Lp(a) levels (≥50 mg/dL) in our cohort. Given the small number of diabetes events, this finding should be interpreted cautiously; however, it is directionally consistent with previous reports suggesting an inverse relationship between Lp(a) concentration and diabetes risk. One proposed explanation is that insulin resistance and hyperinsulinemia may suppress apo(a) production, resulting in lower circulating Lp(a) concentrations, although the underlying mechanisms remain incompletely understood. Vaverková et al. similarly demonstrated inverse correlations between Lp(a) and multiple markers of insulin resistance and metabolic syndrome components, suggesting that elevated Lp(a) may reflect a phenotype characterized by lower, not higher, metabolic dysregulation [13]. Buchmann et al. extended this observation by showing that while an inverse association between metabolic syndrome and Lp(a) was confirmed in men and postmenopausal women, premenopausal women showed a paradoxical positive association—a menopausal status-dependent reversal directly relevant to our cohort and supportive of the null finding reported here [14]. However, findings across studies remain heterogeneous. A recent phenome-wide Mendelian randomization analysis by Larsson et al. reported a weak positive association between genetically predicted Lp(a) levels and type 2 diabetes, highlighting the continuing uncertainty regarding the relationship between Lp(a) and metabolic disease [15]. Furthermore, recent Mendelian randomization data demonstrated no causal association between genetically predicted Lp(a) levels and arterial stiffness despite the well-established role of Lp(a) in ASCVD [16]. Together, these findings suggest that the clinical relevance of Lp(a) may be more specific to atherosclerotic cardiovascular pathways than to broader cardiometabolic or vascular phenotypes.

The cholesterol-dependent and threshold-dependent nature of Lp(a)’s cardiovascular predictive value in women adds further interpretive context. In an analysis combining three large cohorts—the Women’s Health Study (WHS), the Women’s Health Initiative (WHI), and the female participants of the JUPITER trial—Cook et al. demonstrated that Lp(a) predicted incident cardiovascular disease in women only among those with total cholesterol exceeding 220 mg/dL [17]. Among the 94% of JUPITER women with lower cholesterol levels, which approximates the lipid profile of our cohort, hazard ratios were substantially attenuated and became non-significant; in contrast, men in the same trial showed strong associations between Lp(a) and cardiovascular disease irrespective of cholesterol level. Mendelian randomization and observational data from the EPIC-Norfolk cohort similarly indicate that the risk threshold in women for incident coronary artery disease appeared confined to those at or above the 90th Lp(a) percentile—substantially higher than the standard 50 mg/dL threshold—while no such extreme concentration was required in men [18]. Collectively, these data suggest that in premenopausal women without concurrent hyperlipidemia, Lp(a) elevation alone does not appear to be a sufficient driver of either ASCVD or upstream CMD.

The present study complements rather than contradicts the landmark 30-year data from Ridker et al., which established that Lp(a) independently predicts major adverse cardiovascular events in initially healthy American women [19]. Critically, that cohort was predominantly postmenopausal (mean age 54.7 years) and focused exclusively on hard ASCVD endpoints. The present study addresses an earlier and distinct question: whether Lp(a) predicts upstream CMD in relatively young women at low ASCVD risk—precisely those presenting to preventive health check-ups today. Together, the two studies delineate the boundaries of Lp(a)’s clinical utility across the female lifespan. More recently, a dedicated 30-year threshold analysis of the same WHS cohort further underscored that long-term ASCVD risk becomes apparent predominantly at or above 30 mg/dL and is particularly pronounced at very high concentrations (≥120 mg/dL), with no significant association observed among women with LDL-C below the median [20]—a finding consistent with the null cardiometabolic risk observed in our comparably low-risk Korean cohort. Ethnic differences should also be considered when interpreting these findings, as Lp(a) concentrations and their associated cardiovascular risk vary substantially across populations [6,7]. Accordingly, the present results should be interpreted within the context of this Korean female cohort and may not be directly generalizable to other ethnic groups.

In women, these observations are best understood within a life-course framework of Lp(a) [21]. During the reproductive years, estrogen suppresses hepatic apo(a) mRNA transcription, maintaining Lp(a) near its biological nadir. After the menopausal transition, this suppression is lifted and Lp(a) rises substantially, with postmenopausal women exhibiting approximately 17–20% higher concentrations than age-matched men [7,10]. In our cohort, age, BMI, and LDL-C were each independently associated with incident CMD in the fully adjusted model (Table 3), consistent with conventional cardiometabolic risk determinants in a premenopausal population, whereas Lp(a) did not emerge as an independent predictor after adjustment for these established risk factors.

Taken together, these findings carry practical implications for the clinical interpretation of Lp(a) measurements. Current guidelines appropriately recommend Lp(a) measurement at least once in all adults for ASCVD risk assessment [4,5]. In apparently healthy premenopausal women, however, an elevated Lp(a) value should be interpreted principally as a marker of an adverse atherogenic lipid phenotype rather than as an independent predictor of future metabolic deterioration. Its clinical relevance appears to emerge most clearly following the menopausal transition and in the presence of concomitant hyperlipidemia.

This study has several strengths. We analyzed a well-characterized cohort of apparently healthy women with standardized baseline measurements, extended follow-up of up to nearly 18 years, and a substantial subgroup with ≥10 years of observation. The availability of longitudinal clinical data enabled the assessment of incident cardiometabolic outcomes, and consistent findings across multiple analytical approaches—including both categorical and continuous Lp(a) analyses—strengthen the robustness of our conclusions.

Several limitations should be acknowledged. First, this was a single-center study based on a self-referred health screening population. Accordingly, the findings should be interpreted in the context of relatively healthy women undergoing preventive health evaluations. In addition, the study population consisted exclusively of Korean women, which may limit the generalizability of the findings to other populations. Second, no ASCVD events or deaths occurred during follow-up, precluding evaluation of Lp(a) as a predictor of hard cardiovascular endpoints—the outcomes for which its causal role is most firmly established. Third, menopausal status was not available in a complete and standardized format across the entire cohort. Although the cohort was relatively young (median age 41 years), the potential influence of menopause on the observed associations cannot be excluded. Fourth, Lp(a) was measured using a turbidimetric immunoassay and reported in mg/dL rather than nmol/L. In addition, detailed historical calibration information was unavailable. These factors may limit direct comparability with contemporary studies using isoform-independent assays and standardized reporting in nmol/L. Fifth, incident dyslipidemia accounted for the majority of composite CMD events. Accordingly, the composite outcome was driven primarily by lipid-related abnormalities, and caution is warranted when extrapolating these findings to broader cardiometabolic conditions. Sixth, the relatively small number of participants with elevated Lp(a) levels (≥50 mg/dL) may have limited statistical power to detect modest associations, and the possibility of a type II error cannot be excluded. Finally, residual confounding from unmeasured variables cannot be excluded despite multivariable adjustment.

## 5. Conclusions

Elevated Lp(a) is associated with an adverse baseline lipid phenotype but does not independently predict long-term incident CMD in apparently healthy women. These findings suggest that the clinical utility of Lp(a) in younger women may be primarily limited to ASCVD risk assessment rather than broader cardiometabolic risk prediction, and highlight the importance of population- and outcome-specific interpretation of Lp(a) in clinical practice.

## Figures and Tables

**Table 1:** Baseline characteristics according to Lp(a) level in the full screening cohort.

Variable	Total (*n* = 559)	Lp(a) < 50 mg/dL (*n* = 482)	Lp(a) ≥ 50 mg/dL (*n* = 77)	*p*-Value
Age, years	41 [36–46]	41 [36–45]	43 [37–48]	0.081
Body mass index, kg/m^2^	21.6 [19.9–23.4]	21.6 [19.9–23.4]	21.9 [19.9–23.6]	0.550
Current smoking, *n* (%)	7 (1.3)	5 (1.0)	2 (2.6)	0.249
Lp(a), mg/dL	19.0 [10.0–33.0]	16.0 [9.0–25.0]	70.0 [55.0–90.0]	<0.001
Total cholesterol, mg/dL	185 [168–207]	183 [166–205]	194 [176–218]	0.001
LDL-C, mg/dL	105 [89–123]	103 [88–122]	114 [99–139]	0.001
HDL-C, mg/dL	61 [53–72]	61 [53–71]	65 [53–72]	0.442
Triglycerides, mg/dL	76 [56–105]	74 [55–104]	82 [58–116]	0.373
Fasting glucose, mg/dL	81 [76–87]	81 [77–87]	79 [75–86]	0.291
Baseline hypertension, *n* (%)	26 (4.7)	22 (4.6)	4 (5.2)	0.771
Baseline diabetes mellitus, *n* (%)	6 (1.1)	5 (1.0)	1 (1.3)	0.591
Baseline dyslipidemia, *n* (%)	65 (11.6)	49 (10.2)	16 (20.8)	0.012
Baseline CMD *, *n* (%)	83 (14.8)	66 (13.7)	17 (22.1)	0.080

Data are presented as median [interquartile range] or n (%). *p*-values were calculated for comparisons between Lp(a) groups (<50 vs. ≥50 mg/dL). Continuous variables were compared using the Mann–Whitney U test, and categorical variables were compared using the chi-square test or Fisher’s exact test, as appropriate. * Baseline CMD was defined as the presence of at least one of the following: hypertension, diabetes mellitus, or dyslipidemia. Abbreviations: Lp(a), lipoprotein(a); LDL-C, low-density lipoprotein cholesterol; HDL-C, high-density lipoprotein cholesterol; CMD, cardiometabolic disease.

**Table 2:** Baseline characteristics and incident outcomes according to Lp(a) level in the primary longitudinal cohort.

Variable	Total (*n* = 387)	Lp(a) < 50 mg/dL (*n* = 336)	Lp(a) ≥ 50 mg/dL (*n* = 51)	*p*-Value
Baseline Characteristics				
Age, years	41.0 [36.0–45.0]	41.0 [36.0–45.0]	42.0 [37.0–45.5]	0.410
Body mass index, kg/m^2^	21.4 [19.8–23.2]	21.4 [19.8–23.3]	21.2 [19.8–22.4]	0.913
Current smoking, *n* (%)	5 (1.3)	3 (0.9)	2 (3.9)	0.131
Lp(a), mg/dL	19.0 [10.0–32.0]	16.0 [9.0–25.0]	74.0 [56.0–92.0]	<0.001
Total cholesterol, mg/dL	181 [166–200]	180 [164–199]	189 [172–212]	0.026
LDL-C, mg/dL	102 [89–119]	101 [88–118]	107 [96–123]	0.047
HDL-C, mg/dL	63 [55–71]	62 [55–71]	66 [57–73]	0.188
Triglycerides, mg/dL	71 [53–92]	71 [52–92]	73 [55–92]	0.682
Fasting glucose, mg/dL	81 [76–86]	81 [77–86]	78 [75–84]	0.063
Follow-up				
Follow-up duration, years	12.6 [5.0–17.1]	12.6 [5.0–17.1]	12.0 [3.9–17.2]	0.865
Follow-up ≥ 10 years, *n* (%)	224 (57.9)	193 (57.4)	31 (60.8)	0.765
Incident Outcomes				
Hypertension, *n* (%)	30 (7.8)	27 (8.0)	3 (5.9)	0.782
Diabetes mellitus, *n* (%)	16 (4.1)	16 (4.8)	0 (0.0)	0.145
Dyslipidemia, *n* (%)	122 (31.5)	104 (31.0)	18 (35.3)	0.645
Composite CMD *, *n* (%)	130 (33.6)	112 (33.3)	18 (35.3)	0.907

Data are presented as median [interquartile range] or n (%). *p*-values were calculated for comparisons between Lp(a) groups (<50 vs. ≥50 mg/dL). Continuous variables were compared using the Mann–Whitney U test, and categorical variables were compared using the chi-square test or Fisher’s exact test, as appropriate. * Composite CMD was defined as the occurrence of at least one of the following during follow-up: hypertension, diabetes mellitus, or dyslipidemia. Abbreviations: Lp(a), lipoprotein(a); LDL-C, low-density lipoprotein cholesterol; HDL-C, high-density lipoprotein cholesterol; CMD, cardiometabolic disease.

**Table 3:** Cox proportional hazards models for incident composite cardiometabolic disease.

**(A) Primary longitudinal cohort (*n* = 387)**								
Variable	Model 1	*p*	Model 2	*p*	Model 3	*p*	Model 4	*p*
Lp(a) ≥ 50 mg/dL	1.00 (0.61–1.64)	0.986	0.86 (0.52–1.42)	0.558	0.88 (0.53–1.46)	0.616	0.81 (0.49–1.34)	0.405
Age (per 1 year)	–	–	1.07 (1.04–1.11)	<0.001	1.06 (1.03–1.10)	<0.001	1.04 (1.01–1.07)	0.009
BMI (per 1 kg/m^2^)	–	–	–	–	1.08 (1.03–1.14)	0.004	1.05 (1.00–1.11)	0.045
LDL-C (per 1 mg/dL)	–	–	–	–	–	–	1.03 (1.02–1.04)	<0.001
**(B) ≥10-year follow-up subgroup (*n* = 224)**								
Variable	Model 1	*p*	Model 2	*p*	Model 3	*p*	Model 4	*p*
Lp(a) ≥ 50 mg/dL	0.94 (0.55–1.63)	0.835	0.83 (0.48–1.44)	0.502	0.84 (0.49–1.46)	0.501	0.81 (0.46–1.40)	0.443
Age (per 1 year)	–	–	1.06 (1.03–1.10)	<0.001	1.05 (1.01–1.09)	0.008	1.03 (1.00–1.07)	0.081
BMI (per 1 kg/m^2^)	–	–	–	–	1.08 (1.02–1.14)	0.012	1.06 (1.00–1.12)	0.059
LDL-C (per 1 mg/dL)	–	–	–	–	–	–	1.03 (1.02–1.04)	<0.001

Values are hazard ratios (95% confidence intervals). Model 1: unadjusted; Model 2: adjusted for age; Model 3: adjusted for age and BMI; Model 4: adjusted for age, BMI, and LDL-C. Abbreviations: Lp(a), lipoprotein(a); BMI, body mass index; LDL-C, low-density lipoprotein cholesterol.

## Data Availability

The data supporting the findings of this study are not publicly available due to privacy and ethical restrictions governing patient data at Konkuk University Medical Center, but are available from the corresponding author upon reasonable request.

## Supplementary Materials

The following supporting information can be downloaded at: https://www.mdpi.com/article/10.3390/metabo16060390/s1, Table S1: Baseline characteristics and incident outcomes according to Lp(a) level in the ≥10-year follow-up cohort; Table S2: Cox proportional hazards models for incident composite cardiometabolic disease using continuous Lp(a). Figure S1: Kaplan–Meier analyses of individual incident cardiometabolic outcomes according to baseline lipoprotein(a) [Lp(a)] levels.

## Abbreviations

The following abbreviations are used in this manuscript:

ASCVD	Atherosclerotic cardiovascular disease
BMI	Body mass index
CI	Confidence interval
CMD	Cardiometabolic disease
HbA1c	Glycated hemoglobin
HDL-C	High-density lipoprotein cholesterol
HR	Hazard ratio
LDL-C	Low-density lipoprotein cholesterol
LoD	Lower limit of detection
Lp(a)	Lipoprotein(a)
WHS	Women’s Health Study
