# Supplementary material for: Lipoprotein(a) Reflects Baseline Lipid Phenotype but Does Not Predict Long-Term Cardiometabolic Risk in Apparently Healthy Women
Source: Metabolites. 2026 Jun 4;16(6):390. doi: 10.3390/metabo16060390 (PMC13303182; doi:10.3390/metabo16060390)

Supplementary Figure S1. Kaplan–Meier analyses of individual incident cardiometabolic outcomes according to baseline lipoprotein(a) [Lp(a)] levels.

(a) Incident hypertension, (b) Incident diabetes mellitus, (c) Incident dyslipidemia. Participants were stratified according to baseline Lp(a) level (<50 vs. ≥50 mg/dL). Event-free survival was estimated using the Kaplan–Meier method and compared using the log-rank test. Shaded areas represent 95% confidence intervals, and tick marks indicate censored observations. No significant differences were observed for incident hypertension (log-rank $p = 0.56$), diabetes mellitus (log-rank $p = 0.11$), or dyslipidemia (log-rank $p = 0.69$) according to baseline Lp(a) category.

(a) Incident hypertension

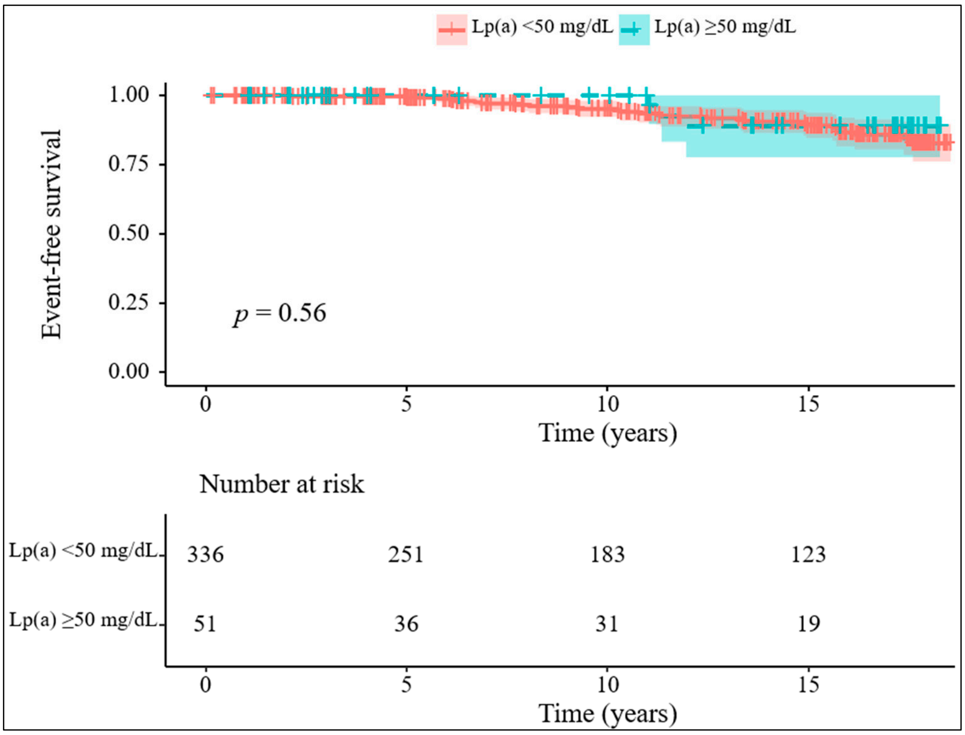

(b) Incident diabetes mellitus

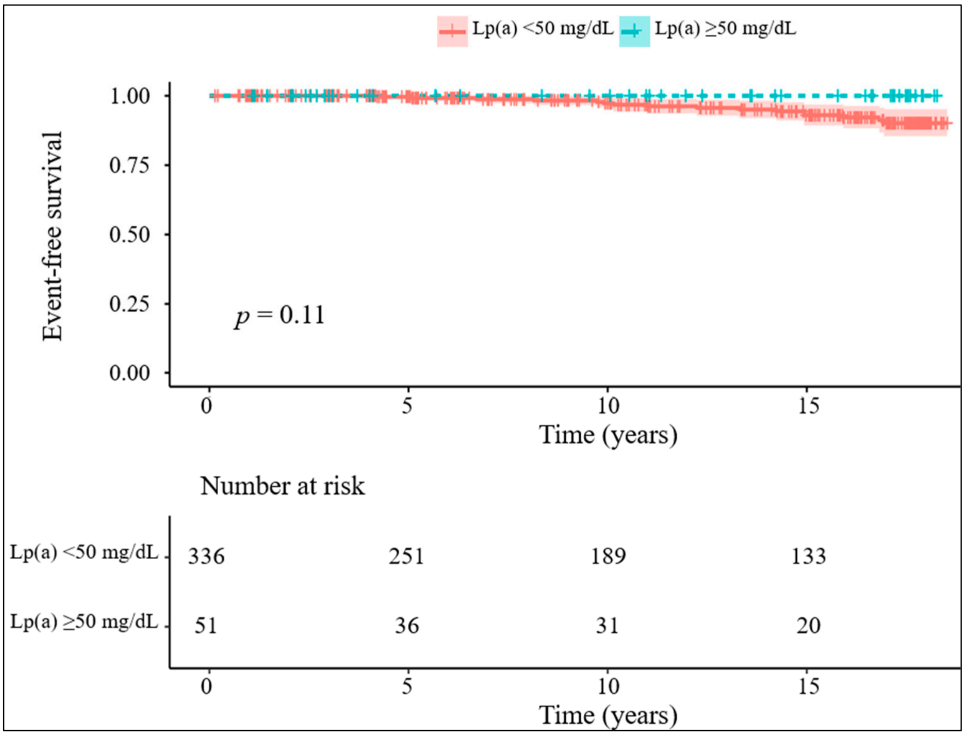

(c) Incident dyslipidemia

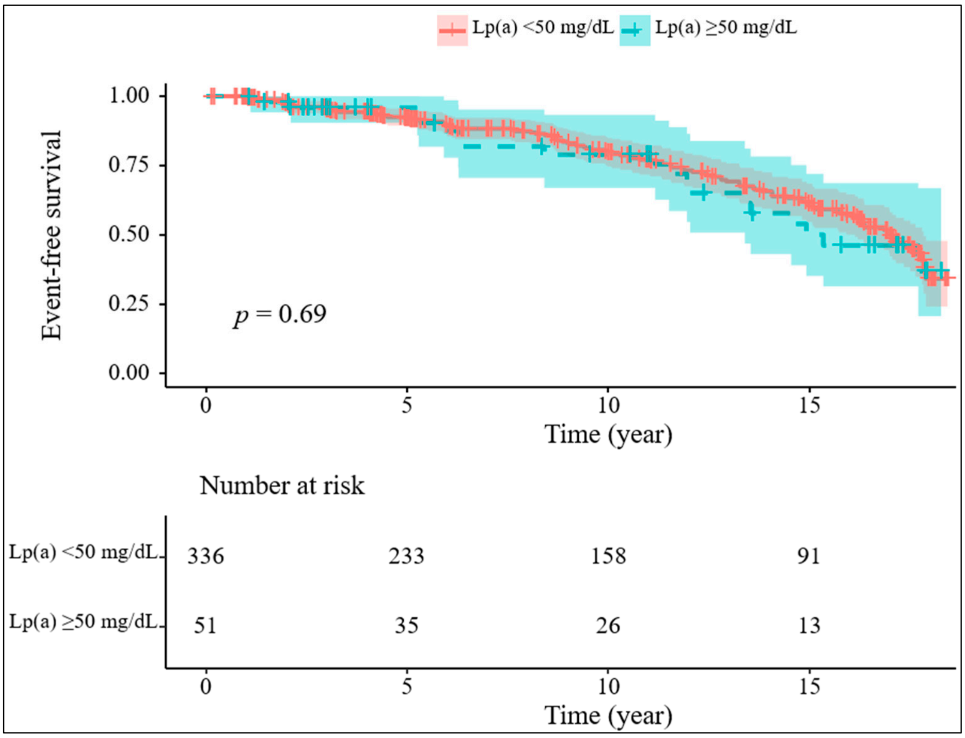

Supplement: Supplementary file 1 [file metabolites-16-00390-s001.zip › Supplementary Figure S1.pdf]
