# Supplementary material for: Lipoprotein(a) Reflects Baseline Lipid Phenotype but Does Not Predict Long-Term Cardiometabolic Risk in Apparently Healthy Women
Source: Metabolites. 2026 Jun 4;16(6):390. doi: 10.3390/metabo16060390 (PMC13303182; doi:10.3390/metabo16060390)
Supplement: Supplementary file 1 [file metabolites-16-00390-s001.zip › Supplementary Table S1.pdf]

Supplementary Table S1. Baseline characteristics and incident outcomes according to Lp(a) level in the ≥10-year follow-up cohort

Variable	Total (n = 224)	Lp(a) <50 mg/dL (n = 193)	Lp(a) ≥50 mg/dL (n = 31)	P-value
**Baseline Characteristics**				
Age, years	41 [37–45]	41.0 [37.0–45.0]	40.0 [37.5–45.0]	0.616
Body mass index, kg/m²	21.0 [19.7–22.9]	21.0 [19.8–22.9]	20.9 [19.4–22.5]	0.700
Current smoking, n (%)	1 (0.4)	1 (0.5)	0 (0.0)	1.000
Lp(a), mg/dL	19.0 [10.0–33.0]	17.0 [9.0–26.0]	74.0 [54.5–92.0]	<0.001
Total cholesterol, mg/dL	182 [169–200]	181 [168–199]	183 [172–204]	0.436
LDL-C, mg/dL	103 [90–119]	103 [89–119]	103 [92–118]	0.705
HDL-C, mg/dL	63 [55–73]	62 [54–72]	66 [58–76]	0.108
Triglycerides, mg/dL	69 [53–90]	69 [53–91]	69 [51–84]	0.468
Fasting glucose, mg/dL	81 [76–85]	81 [76–86]	79 [76–82]	0.287
**Follow-up**				
Follow-up duration, years	16.9 [14.7–17.7]	16.9 [15.0–17.8]	17.1 [13.6–17.5]	0.567
**Incident Outcomes**				
Hypertension, n (%)	29 (12.9)	26 (13.5)	3 (9.7)	0.775
Diabetes mellitus, n (%)	14 (6.2)	14 (7.3)	0 (0.0)	0.227
Dyslipidemia, n (%)	104 (46.4)	89 (46.1)	15 (48.4)	0.967
Composite CMD*, n (%)	110 (49.1)	95 (49.2)	15 (48.4)	1.000

Data are presented as median [interquartile range] or n (%). P-values were calculated for comparisons between Lp(a) groups (<50 vs. ≥50 mg/dL). Continuous variables were compared using the Mann–Whitney U test, and categorical variables were compared using the chi-square test or Fisher's exact test, as appropriate. *Composite CMD was defined as the occurrence of at least one of the following during follow-up: hypertension, diabetes mellitus, or dyslipidemia. Abbreviations: Lp(a), lipoprotein(a); LDL-C, low-density lipoprotein cholesterol; HDL-C, high-density lipoprotein cholesterol; CMD, cardiometabolic disease.
