# Supplementary material for: Lipoprotein(a) Reflects Baseline Lipid Phenotype but Does Not Predict Long-Term Cardiometabolic Risk in Apparently Healthy Women
Source: Metabolites. 2026 Jun 4;16(6):390. doi: 10.3390/metabo16060390 (PMC13303182; doi:10.3390/metabo16060390)
Supplement: Supplementary file 1 [file metabolites-16-00390-s001.zip › Supplementary Table S2.pdf]

Supplementary Table S2. Cox proportional hazards models for incident composite cardiometabolic disease using continuous Lp(a)

(A) Primary longitudinal cohort (*n* = 387)

**Variable**	**Model 1**	***P*-value**	**Model 2**	***P*-value**	**Model 3**	***P*-value**	**Model 4**	***P*-value**
Lp(a) (per 1 mg/dL)	1.00 (1.00–1.01)	0.521	1.00 (0.99–1.01)	0.994	1.00 (0.99–1.01)	0.996	1.00 (0.99–1.01)	0.506
Age (per 1 year)	–	–	1.07 (1.04–1.11)	< 0.001	1.06 (1.03–1.09)	< 0.001	1.04 (1.01–1.07)	0.009
BMI (per 1 kg/m²)	–	–	–	–	1.08 (1.03–1.14)	0.004	1.05 (1.00–1.11)	0.047
LDL-C (per 1 mg/dL)	–	–	–	–	–	–	1.03 (1.02–1.04)	< 0.001

(B) ≥10-year follow-up subgroup (*n* = 224)

**Variable**	**Model 1**	***P*-value**	**Model 2**	***P*-value**	**Model 3**	***P*-value**	**Model 4**	***P*-value**
Lp(a) (per 1 mg/dL)	1.00 (0.99–1.01)	0.755	1.00 (0.99–1.01)	0.812	1.00 (0.99–1.01)	0.803	1.00 (0.99–1.01)	0.488
Age (per 1 year)	–	–	1.06 (1.03–1.10)	< 0.001	1.05 (1.01–1.09)	0.009	1.03 (1.00–1.07)	0.080
BMI (per 1 kg/m²)	–	–	–	–	1.08 (1.02–1.14)	0.011	1.06 (1.00–1.12)	0.061
LDL-C (per 1 mg/dL)	–	–	–	–	–	–	1.03 (1.02–1.04)	< 0.001

Values are hazard ratios (95% confidence intervals). Model 1: unadjusted; Model 2: adjusted for age; Model 3: adjusted for age and BMI; Model 4: adjusted for age, BMI, and LDL-C. Abbreviations: Lp(a), lipoprotein(a); BMI, body mass index; LDL-C, low-density lipoprotein cholesterol.
